# Phototaxis of the Unicellular Red Alga *Cyanidioschyzon merolae* Is Mediated by Novel Actin-Driven Tentacles

**DOI:** 10.3390/ijms21176209

**Published:** 2020-08-27

**Authors:** Sascha Maschmann, Karin Ruban, Johanna Wientapper, Wilhelm J. Walter

**Affiliations:** Institute for Plant Science and Microbiology, University of Hamburg, 20146 Hamburg, Germany; sascha.maschmann@ugent.vib.be (S.M.); karin.ruban@cssb-hamburg.de (K.R.); johanna.wientapper@studium.uni-hamburg.de (J.W.)

**Keywords:** *Cyanidioschyzon merolae*, rhodophyta, phototaxis, actin, tentacles

## Abstract

Phototaxis, which is the ability to move towards or away from a light source autonomously, is a common mechanism of unicellular algae. It evolved multiple times independently in different plant lineages. As of yet, algal phototaxis has been linked mainly to the presence of cilia, the only known locomotive organelle in unicellular algae. Red algae (Rhodophyta), however, lack cilia in all stages of their life cycle. Remarkably, multiple unicellular red algae like the extremophile *Cyanidioschyzon merolae* (*C. merolae*) can move towards light. Remarkably, it has remained unclear how *C. merolae* achieves movement, and the presence of a completely new mechanism has been suggested. Here we show that the basis of this movement are novel retractable projections, termed tentacles due to their distinct morphology. These tentacles could be reproducibly induced within 20 min by increasing the salt concentration of the culture medium. Electron microscopy revealed filamentous structures inside the tentacles that we identified to be actin filaments. This is surprising as *C. merolae*’s single actin gene was previously published to not be expressed. Based on our findings, we propose a model for *C. merolae*’s actin-driven but myosin-independent motility. To our knowledge, the described tentacles represent a novel motility mechanism.

## 1. Introduction

Phototaxis is the ability of an organism to autonomously move towards or away from a light source. It has evolved multiple times independently in different plant lineages and is a widespread acquirement of unicellular algae [1]. Phototactic unicellular and colonial green and brown algae use flagella or cilia to swim in helical trajectories, like most phototactic eukaryotic organisms [2,3]. The best-characterized phototaxis model, the green alga *Chlamydomonas reinhardtii*, propells itself via the synchronous breaststroke beating of its flagellar pair. The underlying mechanisms of light perception and steering are well understood [4]. The common molecular basis of flagella and cilia is the so-called axoneme which consists of a characteristic arrangement of nine microtubule doublets. Dynein motor proteins generate sliding motions between adjacent microtubules which are integrated into a beating or rotational motion [5].

Red algae (Rhodophyta), however, lack flagella and cilia in all stages of their life cycle [1]. Remarkably, positive phototaxis by the unicellular Rhodophyta *Porphyridium cruentum* [6], *Porphyridium purpureum*, *Rhodella maculata*, *Dixoniella grisea* [7], *Timspurckia oligopyrenoides*, and *Erythrolobus madagascarensis* [8] was observed when exposed to unilateral light. Even in the acidophilic, thermophilic unicellular Cyanidales, a sister group to all other red algae, phototaxis was observed in *Cyanidioschyzon merolae* (*C. merolae*) and *Cyanidium caldarium* [9]. Surprisingly, no external appendages were observed to be involved in cell movement. Consequently, the presence of a completely new motility mechanism was suggested [9]. We combined molecular biological, biochemical, and imaging techniques to analyse the structural basis of *C. merolae*’s phototactic movement.

## 2. Results and Discussion

**Figure 1:**
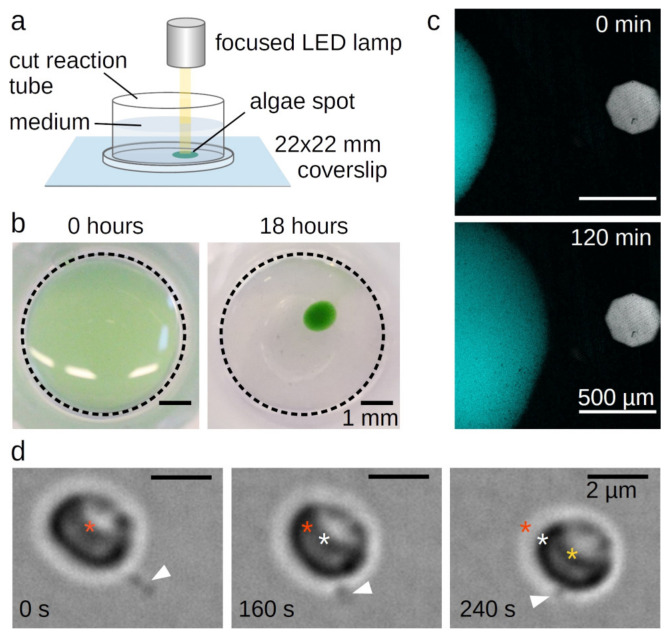
Phototaxis of *Cyanidioschyzon merolae*. (**A**) Set-up for the light microscopical observation of *C. merolae*’s phototaxis. (**B**) Sedimented *C. merolae* culture before and after condensing at a focused light spot. (**C**) Movement of condensed *C. merolae* culture towards a focused light spot. The figure shows a combined autofluorescent signal of the chloroplasts excited at 640 nm (cyan) and a bright field image indicating the position of the focused light spot (grey). (**D**) Single *C. merolae* cell moving by attaching the tip of its protrusion (white arrowhead) to the surface and then retracting. Asterisks mark the center of the cell at the timepoint of attachment (red), after 160 s (white), and after 240 s (yellow).

We found that sedimented cells of a liquid *C. merolae* culture condensed at a focused light spot within 18 h (Figure 1a,b). Similar behaviour was previously described for *C. merolae* and *Cyanidium caldarium* [9] as well as for *Porphyridium cruentum* [6]. Upon moving the light, the condensed cells immediately followed the spot with a velocity of approximately 2.5 µm⸱min^−1^ (Figure 1c, Supplementary Movie S1). This movement is considerably slower than swimming phototactic algae like *Chlamydomonas reinhardtii* [10]. Swimming cells are propelled by microtubule-based flagella or cilia [11] that are lacking in all stages of the Rhodophyta’s life cycle. Some cells, however, can slowly glide over surfaces using pseudopodia facilitated by actin filament dynamics at the cell periphery [12].

*C. merolae*’s genome contains a single actin gene [13,14]. Nevertheless, no actin cDNA clones could be obtained [13] nor was it detected by fluorescence microscopy with FITC-phalloidin, which specifically binds actin filaments, or by transmission electron microscopy in dividing cells [15]. Consequently, Ohnuma et al. proposed a “non-conventional system” to enable *C. merolae*’s movement [9]. Looking at single moving cells, we found that this “system” consists of single or few long protrusions. Cells attach the tip of the protrusions to a surface and move forward via retraction (Figure 1d, Supplementary Movie S2). As these single stepping events were recorded in bright-field microscopy in absence of light stress, the observed stepping velocity was lower than the leading edge of the phototactic cell spot in Figure 1c.

**Figure 2:**
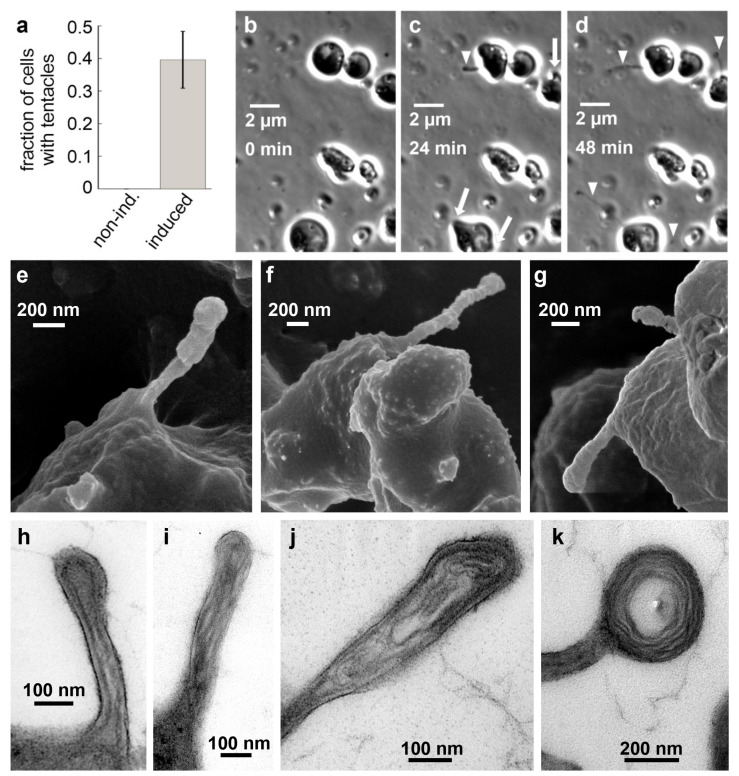
Filopodia-like tentacles in salt-induced *C. merolae* cells. (**A**) Fraction of cells in suspension culture with tentacles before (0 %, N_tentacles_ = 0, N_total_ = 176, n = 4) and after addition of 50 mM NaCl (39.6 ± 8.7 %, N_tentacles_ = 69, N_total_ = 178, n = 4). (**B**–**D**) Time series of *C. merolae* cells growing filopodia-like projections (arrowheads) from bulbs (arrows) upon induction with 50 mM sodium chloride imaged in phase-contrast microscopy. (**E**–**G**) SEM images show the presence of the extended filopodia-like structures that we termed tentacles. (**H**–**K**) Negative staining TEM images of thin sections through *C. merolae* tentacles show the presence of long filamentous structures that are oriented in a parallel fashion in the tentacle shaft (**H**–**I**) and in concentric circles in the tentacle head (**J**–**K**).

Under optimal growth conditions, *C. merolae* does not form the observed projections, which could explain why they have gone unnoticed until now. Low light stress induces the formation of projections, yet complicates the observation as well as biochemical and molecular biological bulk assays. Therefore, we tested whether alternative stress factors have a similar effect. We found that the addition of 50 mM sodium chloride to the culture medium highly reproducibly induced the growth of mostly one but sometimes up to three projections identical to those formed under light stress in about 40% of all observed cells within 60 min (Figure 2a–d, Supplementary Movie S3). The formation of the projections can be divided into two steps. Initially, a bulb appears on the cell’s surface. Subsequently, a thin projection bursts from that bulb, on which the bulb quickly disappears. The protrusions grow up to a multiple cell length. Using scanning electron microscopy (SEM), we obtained a more detailed view of the projections’ structure. SEM images show a shank with a diameter of ~100 nm and an expanded head (Figure 2e–g). We performed transmission electron microscopy (TEM) to get insight into the inner structure of the projections. We found filamentous structures with parallel orientation in the shank and circular orientation in the head (Figure 2h–k). The parallel filament orientation in the shank is highly reminiscent of the parallel orientation of actin filaments in filopodia used by certain animal cells [16]. The circular filament orientation in the head, however, is quite distinct from filopodia and most likely facilitates a sucker-like attachment to surfaces by a contraction of the rings. Based on their overall structure and observed function, we decided to term the protrusions “tentacles”. 

Due to the structural resemblance of the observed filaments with F-actin, we decided to re-investigate the expression of *C. merolae*’s actin and actin-related proteins. By alignment of protein sequences using the BLAST algorithm, we identified five actin-related proteins with a sequence identity of 24 to 51% (Table 1). 

**Figure 3:**
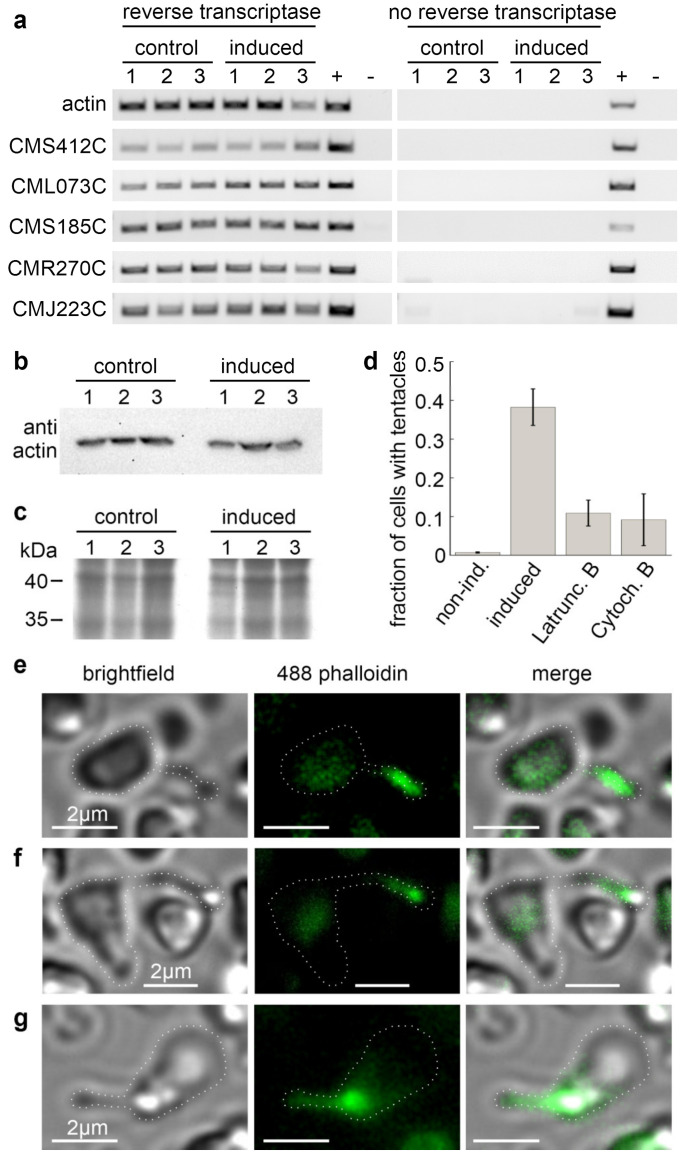
The expression of actin and actin-related proteins in *C. merolae*. (**A**) RT-PCR of the genes of actin and actin-related proteins before and after tentacle induction. (**B**) Western blot of monoclonal anti-actin antibody clone 10-B3 (Sigma-Aldrich, St. Louis, MO, USA). (**C**) SDS-PAGE of the protein samples used in (**B**). (**D**) Treatment of salt-induced cells with the actin cytoskeleton-disrupting drugs Latrunculin B or Cytochalasin B reduced the fraction of observed cells with tentacles from 38.2 ± 4.7 % (N_tentacles_ = 70, N_total_ = 185, n = 4) in DMSO-treated control samples to 10.9 ± 3.3 % (N_tentacles_ = 23, N_total_ = 214, n = 4) and 9.2 ± 6.7% (N_tentacles_ = 14, N_total_ = 178, n = 4), respectively. DMSO-treated, non-induced cells showed no tentacle formation (0.7 ± 1.5 %, N_tentacles_ = 1, N_total_ = 148, n = 5). (**E**–**G**) Tentacles are positive for staining with Alexa Fluor 488-phalloidin. Brightfield, fluorescence, and merged images of three exemplary cells. Dotted white lines highlight the cell outlines.

A reverse transcriptase PCR (RT-PCR) with three non-treated and three induced *C. merolae* cultures showed an expression of actin and all actin-related genes independent of the induction (Figure 3a). As this contradicts previous claims on actin expression in *C. merolae* [13,15], we performed a Western blot with a monoclonal actin antibody that confirmed our RT-PCR results (Figure 3b,c). Remarkably, the claims on the non-expression of actin were mainly based on the absence of actin filaments in electron microscopical observations of cytokinesis and the lack of cDNA clones. Yet, F-actin might only be present in the previously unknown tentacles, and cDNA libraries are not always complete.

As we could demonstrate the expression of actin in general, we analysed the tentacle formation in the presence of the actin cytoskeleton-disrupting drugs. We found that the fraction of cells forming tentacles within one hour after addition of 50 mM NaCl dropped from 38.2 ± 4.7% in DMSO-treated control samples to 10.9 ± 3.3% in cells treated with 10 µM Latrunculin B, and to 9.2 ± 6.7% in samples treated with 3.3 µM Cytochalasin B (Figure 3d). Moreover, we stained fixated, salt-induced cells with Alexa Fluor 488 phalloidin. The fluorescence signals co-localised with the tentacles (Figure 3e–g). Combined, these findings strongly support our hypothesis that actin polymerisation drives the tentacle formation. Remarkably, the unchanged levels in actin expression between non-treated and induced cells indicate that tentacle formation in stressed *C. merolae* cells relies on a permanently present actin pool. This pool could explain the rapid formation of tentacles within the first few minutes after induction (Figure 2b–d). 

The tentacles resemble filopodia. Filopodia formation is the result of an orchestrated interplay between actin filaments and multiple well-studied factors including the motor activity of myosins, actin remodelling factors including the ARP2/3 complex, crosslinking proteins such as fascin, α-actinin, or formins, signalling factors like Rho GTPases, and the membrane-deforming activity of members of the I-BAR family. However, analogues for most of these proteins that are essential for the formation of filopodia are missing in the C. merolae genome (Table 2). This finding suggests that, despite their initial resemblance, tentacles are both mechanistically and evolutionarily distinct from filopodia.

Filopodia tend to emerge smoothly from lamellipodia, which are sheet-like structures based upon a branched actin filament network [31]. In contrast, we observed that the tentacles burst from a previously formed large bulb (Figure 2a). The bulb presumably results from growing actin filaments pushing against a larger plasma membrane area. Unlike in filopodia, the filaments are not guided and assisted by membrane-shaping I-BAR proteins. However, for microtubules, it was shown that the accumulation of crosslinking proteins at the membrane–microtubule interface combined with microtubule polymerisation suffices to effectively drive membrane tubulation [32]. 

More surprising in the context of an actin-based motile system is the complete absence of myosin motor genes in *C. merolae*’s genome [14]. However, previous studies on filopodia have demonstrated that pulling forces can be exerted independently from myosin. The rearward-directed actin flow that results from F-actin depolymerization in the filopodia tip, together with inward forces arising from membrane tension, is sufficient to generate forces in the low pN range [19,20]. Similarly, the putative attachment of the tentacle head to the surface via contraction of its circular actin structures does not rely on the function of myosins. Recent studies showed a myosin-independent contraction of actin filament rings by the cross-inking protein anillin [33] *C. merolae*’s genome does not code for anillin. Nevertheless, other crosslinkers are present and might be able to drive a similar mechanism (Table 2).

**Figure 4:**
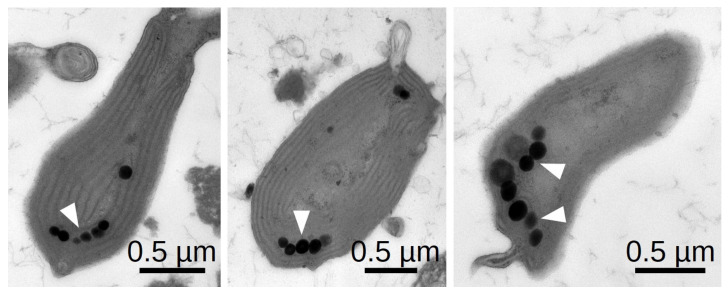
Negative staining TEM of thin sections through three exemplary *C. merolae* cells showing the typical arrangement of lipid droplets.

Besides the mechanical ability to move given by *C. merolae*’s tentacles, phototaxis requires a sensing mechanism that enables an oriented movement response with respect to the direction and intensity of incident light. Other phototactic microalgae like *Chlamydomonas reinhardii* and *Euglena gracilis* can sense the direction of light through a specialised light-sensitive organelle called the eyespot. The eyespot primarily consists of a photoreceptor combined with an arrangement of lipid droplets. The modulation of the light intensity resulting from the respective orientation of the receptor to the lipid droplets allows the cell to adapt its trajectory [34]. Electron microscopy images show that *C. merolae* has multiple arranged lipid droplets that potentially serve to sense light direction (Figure 4). They are less arranged than the eyespot of *Chlamydomonas reinhardtii,* which might be due to the significantly smaller cell size and velocity of *C. merolae*. While in green algae, a rhodopsin pigment mediates phototaxis [34], and no rhodopsin-like genes were identified in the *C. merolae* genome. However, several genes coding for blue-light-sensing cryptochromes were found [35]. As *C. merolae* requires a sensing mechanism for phototaxis, it is a fair assumption that the observed lipid droplets play the described role of modulating light intensity.

**Figure 5:**
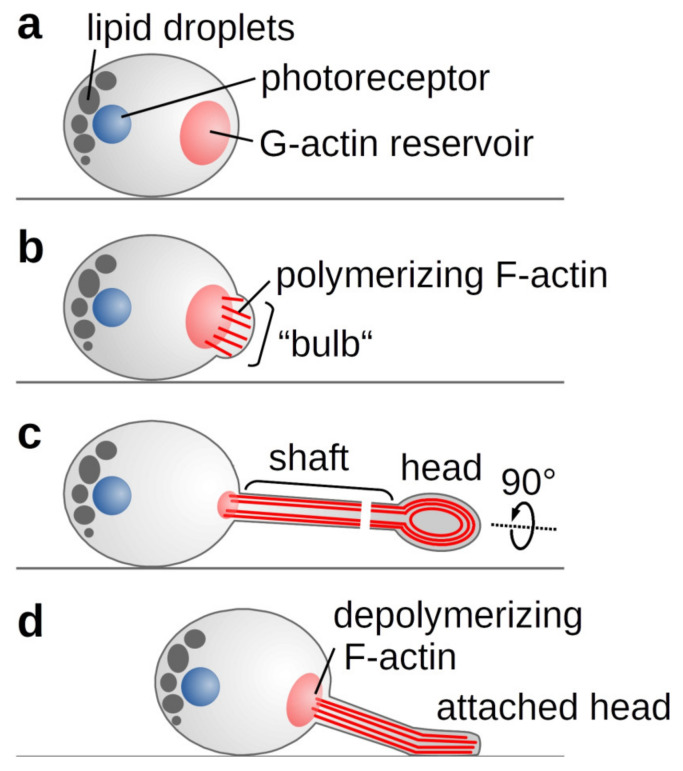
Schematic representation of *C. merolae*’s phototactic mechanism. (**A**) Light or salt stress trigger actin polymerisation from an existing pool of G-actin. (**B**) The growing filaments push against the plasma membrane forming the observed bulb. (**C**) As membrane-shaping I-BAR proteins are missing, a random F-actin bundle prevails to form the growing tentacle. In the tentacle’s shaft, parallel actin filaments drive the elongation, whereas contraction of circular filaments in the tentacle head allows attachment to a surface. (**D**) Actin depolymerisation and membrane tension enable the retraction of the tentacle.

Based on our findings, we propose a novel myosin-independent model for phototaxis in *C. merolae* (Figure 5). The directionality of *C. merolae*’s movement is governed by a combination of a photoreceptor and an arrangement of lipid droplets.

To our knowledge, the observed tentacles represent a completely novel organelle for motility. The previously reported two-dimensional phototaxis in other red algae, however, hints towards tentacles being a more general but largely overlooked feature in unicellular Rhodophyta.

## 3. Methods

### 3.1. Culture Conditions and Tentacle Induction

The *C. merolae* strain 10D was cultivated in acidic Allen medium (20 mM (NH_4_)2SO_4_, 4 mM KH_2_PO_4_, 2 mM MgSO_4_, 1 mM CaCl_2_, 0.3 µM FeCl_3_, 0.7 MnCl_2_, 0.3 µM ZnSO_4_, 70 nM CoCl_2_, 40 nM Na_2_MoO_4_, 10 µM Na_2_EDTA, adjusted to pH 2.3 with H_2_SO_4_) on a heate multiposition magnetic stirrer (RO 15, IKA, Staufen, Germany) at 300 rpm and 43 °C in a 12h/12h day/night cycle with a light intensity of 25 µmol⸱m^−2^.

In suspension cultures, no cells with tentacles were observed. The addition of 50 mM NaCl induced tentacles. First tentacle formation could be observed after about 15 min. After 60 min, 39.6 ± 8.7% of cells had formed tentacles (Figure 2a). Control cells were treated with the same volume of water instead of a NaCl solution. The means and SD of the fraction of cells with tentacles were calculated from five independent samples each.

### 3.2. Inhibitor Treatment

To perform assays with pH-sensible drugs, the pH of the used cultures were adjusted to 7.0 with KOH directly before the drug treatment. Control samples were treated equally. Four independent suspension culture samples were supplemented with 3.3 µM Cytochalasin B or 10 µM Latrunculin B, both dissolved in DMSO 10 min prior to induction of tentacle growth with 50 µM NaCl. Induced and non-induced control samples were supplemented with 2% DMSO to rule out effects of the solvent. Samples were sedimented on a coverslip, and the fraction of cells with tentacles (N_tentacles_/N_total_) was determined in bright-field microscopy. The means and SD of the fraction of cells with tentacles were calculated from the independent samples.

### 3.3. Actin Staining

Samples of a salt-induced *C. merolae* culture were fixated in formaldehyde (4%) for 30 min. Actin filaments were stained for 30 min by addition of 0.1 µM Alexa Fluor 488-phalloidin (A12379, Thermo Fisher Scientific, Waltham, Massachusetts, USA). Cells were gently pelleted and resuspended in Allen medium prior to analysis in light microscopy.

### 3.4. Light Microscopy

Images were acquired by the NIS software packages (Nikon, Tokio, Japan) using an sCMOS camera (Andor Zyla 4.2, Oxford Instruments, Abingdon, UK) mounted on an inverted fluorescence microscope equipped with an autofocus system (Eclipse Ti, Nikon, Tokio, Japan). Phase-contrast microscopy images were recorded with the ProgRes CT5 (Jenoptic, Jena, Germany). Cells were visualised using bright field, phase contrast, or fluorescence epi-illumination with the respective filter sets.

### 3.5. Transmission Electron Microscopy

Samples of an induced *C. merolae* culture were gently pelleted and resuspended in cacodylate buffer (75 mM cacodylate, pH 7.0) supplemented with 2% glutaraldehyde. Samples were fixed for 3.5 h on ice. Cells were immobilised in 2% agarose in cacodylate buffer before fixing with 1% OsO4 overnight at 4 °C. After washing with cacodylate buffer three more times, samples were dehydrated through a graded series of acetone in cacodylate buffer (30, 50, 70, 90, 100%) at 4 °C with two additional changes in the 100% at room temperature. Cells were embedded into epoxy resin (ERL-4221D, D.E.R 736, nonenyl succinic anhydride, dimethylaminoethanol) following a protocol by Spurr [36].

Ultra-thin sections (~80 nm) were prepared on a microtome equipped with a diamond knife (Ultracut E, Reichert-Jung, Heidelberg, Germany) and transferred to copper grids (150 mesh) with a film of polyvinyl butyral (Mowital). The probes were contrasted with solutions of 2% uranyl acetate and 2% lead citrate for 10 min each. Images were acquired at 100kV on a transmission electron microscope (LEO 906E, Zeiss, Jena, Germany) equipped with a CCD camera (MultiScan 794, Gatan, Pleasanton, CA, U.S.A.).

### 3.6. Scanning Electron Microscopy

For scanning electron microscopy, samples of a salt-induced *C. merolae* culture were fixated in formaldehyde (1%), dehydrated through an ascending series of ethanol (30, 50, 70, 90, 100%), and dried at the critical point with Balzers CPD 030 Critical Point Dryer (BALTEC, Pfäffikon, Switzerland). After coating samples with gold using a sputter coater (SCD 050, BAL-TEC), scanning electron micrographs were taken with a LEO 1525 (Zeiss, Jena, Germany).

### 3.7. Gene Expression Analysis

Three induced and three non-induced control samples were frozen in liquid nitrogen and disrupted in a bead mill (Tissue Lyser Qiagen, Retsch GmbH, Haan, Germany). Total RNA was extracted from the samples using a purification kit (NucleoSpin, Macherey-Nagel, Düren, Germany). For positive controls, samples were not treated with DNaseI. cDNA synthesis was performed using a reverse transcription kit (QuantiTect, QIAGEN, Hilden, Netherlands). PCR was conducted using total cDNA as template, gene-specific primers (Table 3), and Q5 polymerase (New England Biolabs, Ipswich, Massachusetts, U.S.A) according to manufacturer instructions. Negative controls did not contain template cDNA.

### 3.8. SDS-PAGE and Immunoblotting

Three induced and three non-induced control samples were harvested by centrifugation (2500× *g*, 4 °C, 60 s). Subsequently, the cells were resuspended in extraction buffer (120mM Tris, pH 6.8, 2% SDS, 16% glycerol, 0.01% Bromphenol blue, 20mM DTT, 0.01% Triton X-100 2×PBS) and heated for ten minutes at 96 °C. Cell debris was pelleted (21,000× *g*, 21 °C, 5 min), and supernatants were loaded to hand-cast polyacrylamide gels (stacking gel 5% polyacrylamide, 0.1% SDS; resolving gel 10% polyacrylamide, 0.1% SDS). The proteins were stacked at 90 V for five minutes and subsequently separated at 130 V for 45 min.

Coomassie staining was conducted according to Dyballa and Metzger [37].

For immunoblotting, proteins were semi-dry blotted to a PVDF membrane at 3.25 A·cm^-2^ for 25 min. The membrane was then blocked for 1 h in blocking solution (1×TBS-T (50 mM Tris, 150 mM NaCl, 0.05% Tween-20, pH 7.5), 2% skim milk powder). The primary anti-actin antibody clone 10-B3 (Sigma-Aldrich, St. Louis, Missouri, USA ) was diluted 1:5000 in blocking solution and applied to the membrane, then slowly shaken at 4 °C for 1 h. After subsequent washing in TBS-T, the secondary anti-mouse IgG peroxidase antibody (Sigma-Aldrich, USA) diluted 1:5000 in blocking solution was applied for 1 h at 4 °C. Afterwards, the membrane was washed in TBS-T, and subsequently the secondary antibody was detected by adding chemiluminescence solution (90mM Tris, 227 µg·mL^−1^ luminol, 2.06 mg·mL^−1^ trans-4-hydroxycinnamic acid, 0.014% H_2_O_2_). The chemiluminescence was detected with the ChemiDoc Touch Imaging System (BioRad Laboratories, Hercules, California, U.S.A.). 

### 3.9. Bioinformatics 

The identification of *C. merolae* analogous of actin and actin-like proteins in Table 1 and filopodia-related proteins in Table 2 was performed in a two-step process. In the first step, we searched the NCBI database (https://www.ncbi.nlm.nih.gov) for annotated analogues in the *C. merolae* 10D (NCBI taxid: 280699) genome. Subsequently, we performed a protein BLAST search [38] based on 5-10 known sequences of the proteins in question from other species with a cutoff of E<e^-5^.

## Figures and Tables

**Table 1:** Percent identity matrix of actin and the actin-related proteins from *C. merolae*.

	1	2	3	4	5	6
**1**: CMM237C actin	100	50.93	23.78	28.57	31.01	36.17
**2**: CMS412C		100	25.17	27.20	29.48	31.61
**3**: CMJ223C			100	21.02	27.02	27.24
**4**: CMS185C				100	23.32	23.70
**5**: CML073C					100	27.89
**6**: CMR270C						100

**Table 2:** Proteins involved in filopodia formation and their analogues in *C. merolae.*

	Protein	*C. merolae* Analogue
	actin	CMM237C
	myosin-X [17,18]	none
Actin remodelling factors	actin-related proteins [12]	CMS412C, CMJ223C, CMS185C, CML073C, CMR270C
	other ARP2/3 complex proteins (p16, p20, p21, p34, and p40) [12]	none
	cofilin	CMN147C
	profilin	CMP220C
	villin	none
	thymosin	none
	capping protein CP [19]	none
	gelsolin [20]	none
F-actin-crosslinking proteins	diaphanous-related formin-2 [21,22]	CMN212C
	formin	CMN049C
	fascin [23,24]	CMD091C
	anillin	none
Rho GTPases and other signalling factors	CDC42 [25]	CMJ091C, CMH183C, and multiple putative candidates
	RIF, Rho in filopodia [26]	multiple putative candidates
	WASP/WAVE [27]	none
	ENA/VASP [24,28]	none
	LPR1, lipid phosphatase-related protein-1 [29]	none
I-BAR proteins [30]	insulin receptor substrate p53	none
	MIM	none
	ABBA	none
	IRTKS	none

**Table 3:** List of primers used for RT-PCR.

	Forward Primer	Reverse Primer
CMM237C:	ATTGGGAGTGAGCGTTTCAG	ATTTGCGGTGTACCACAGAG
CMS412C:	CCGTATGGTCGCATCTCTTC	GCTGCCAAGTCTGTTAGGTC
CMJ223C:	TGCTGGCTTTGCGGGTAGTG	AGTTCTTGGCGGGTCTCCTG
CMS185C:	GCGGACGGCTCCTGAATAAC	GACTCGGGTGCGAGGAAATG
CML073C:	ATTGGGCAGAGTCAACGAAG	TTTAGTACGCGCCAGTCAAG
CMR270C:	GGCGTTGTTCTCGACTGTGG	CGTCAAGCGGCTCTATTCGG

## Supplementary Materials

Supplementary materials can be found at: https://www.mdpi.com/1422-0067/21/17/6209/s1.

## References

[1] Jékely G. (2009). Evolution of phototaxis. Philos. Trans. R. Soc. B Biol. Sci..

[2] Ueki N., Matsunaga S., Inouye I., Hallmann A. (2010). How 5000 independent rowers coordinate their strokes in order to row into the sunlight: Phototaxis in the multicellular green alga volvox. BMC Biol..

[3] Kinoshita N., Nagasato C., Motomura T. (2017). Phototaxis and chemotaxis of brown algal swarmers. J. Plant Res..

[4] Bennett R.R., Golestanian R. (2015). A steering mechanism for phototaxis in chlamydomonas. J. R. Soc. Interface.

[5] Ishikawa T. (2016). Axoneme structure from motile cilia. Cold Spring Harb. Perspect. Biol..

[6] Nultsch W., Schuchart H. (1980). Photomovement of the red alga *Porphyridium Cruentum* (Ag.) Naegeli. Arch. Microbiol..

[7] Pickett-Heaps J., West J., Wilson S., McBride D. (2001). Time-lapse videomicroscopy of cell (spore) movement in red algae. Eur. J. Phycol..

[8] Yang E.C., Scott J., West J.A., Orlova E., Gauthier D., Küpper F.C., Yoon H.S., Karsten U. (2010). New taxa of the Porphyridiophyceae (Rhodophyta): Timspurckia oligopyrenoides gen. et sp. nov. and erythrolobus madagascarensis sp. nov. Phycologia.

[9] Ohnuma M., Misumi O., Kuroiwa T. (2011). Phototaxis in the unicellular red algae *Cyanidioschyzon merolae* and *Cyanidium caldarium*. Cytologia.

[10] Fujita S., Matsuo T., Ishiura M., Kikkawa M. (2014). High-throughput phenotyping of Chlamydomonas swimming mutants based on nanoscale video analysis. Biophys. J..

[11] Wan K.Y. (2018). Coordination of eukaryotic cilia and flagella. Essays Biochem..

[12] Pollard T.D., Borisy G.G. (2003). Cellular motility driven by assembly and disassembly of actin filaments. Cell.

[13] Takahashi H., Takano H., Yokoyama A., Hara Y., Kawano S., Toh-E A., Kuroiwa T. (1995). Isolation, characterization and chromosomal mapping of an actin gene from the primitive red alga *Cyanidioschyzon Merolae*. Curr. Genet..

[14] Matsuzaki M., Misumi O., Shin-I T., Maruyama S., Takahara M., Miyagishima S.-Y., Mori T., Nishida K., Yagisawa F., Nishida K. (2004). Genome sequence of the ultrasmall unicellular red alga *Cyanidioschyzon merolae*. 10D Nature.

[15] Suzuki K., Kawazu T., Mita T., Takahashi H., Itoh R., Toda K., Kuroiwa T. (1995). Cytokinesis by a contractile ring in the primitive red alga cyanidium caldarium RK-1. Eur. J. Cell Biol..

[16] Medalia O., Beck M., Ecke M., Weber I., Neujahr R., Baumeister W., Gerisch G. (2007). Organization of actin networks in intact filopodia. Curr. Biol..

[17] Bohil A.B., Robertson B.W., Cheney R. (2006). Myosin-X Is a molecular motor that functions in filopodia formation. Proc. Natl. Acad. Sci. USA.

[18] Tokuo H., Mabuchi K., Ikebe M. (2007). The motor activity of myosin-x promotes actin fiber convergence at the cell periphery to initiate filopodia formation. J. Cell Biol..

[19] Sinnar S.A., Antoku S., Saffin J.-M., Cooper J.A., Halpain S. (2014). Capping protein is essential for cell migration in vivo and for filopodial morphology and dynamics. Mol. Biol. Cell.

[20] Lu M., Witke W., Kwiatkowski D.J., Kosik K.S. (1997). Delayed retraction of filopodia in gelsolin null mice. J. Cell Biol..

[21] Yang C., Czech L., Gerboth S., Kojima S.-I., Scita G., Svitkina T.M. (2007). Novel roles of formin mdia2 in lamellipodia and filopodia formation in motile cells. PLoS Biol..

[22] Schirenbeck A., Bretschneider T., Arasada R., Schleicher M., Faix J. (2005). The diaphanous-related formin dDia2 is required for the formation and maintenance of filopodia. Nat. Cell Biol..

[23] Vignjevic D.M., Kojima S.-I., Aratyn Y., Danciu O., Svitkina T., Borisy G.G. (2006). Role of fascin in filopodial protrusion. J. Cell Biol..

[24] Harker A.J., Katkar H.H., Bidone T.C., Aydin F., Voth G.A., Applewhite D.A., Kovar D.R. (2019). Ena/VASP processive elongation is modulated by avidity on actin filaments bundled by the filopodia cross-linker fascin. Mol. Biol. Cell.

[25] Nobes C.D., Hall A. (1995). Rho, Rac, and Cdc42 GTPases regulate the assembly of multimolecular focal complexes associated with actin stress fibers, lamellipodia, and filopodia. Cell.

[26] Pellegrin S., Mellor H. (2005). The Rho Family GTPase Rif induces Filopodia through MDia2. Curr. Biol..

[27] Stradal T.E., Scita G. (2006). Protein complexes regulating Arp2/3-mediated actin assembly. Curr Opin Cell Biol..

[28] Krause M., Dent E.W., Bear J.E., Loureiro J.J., Gertler F.B. (2003). Ena/VASP proteins: regulators of the actin cytoskeleton and cell migration. Annu. Rev. Cell Dev. Biol..

[29] Sigal Y.J., Quintero O.A., Cheney R.E., Morris A.J. (2007). Cdc42 and ARP2/3-independent regulation of filopodia by an integral membrane lipid-phosphatase-related protein. J. Cell Sci..

[30] Scita G., Confalonieri S., Lappalainen P., Suetsugu S. (2008). IRSp53: Crossing the road of membrane and actin dynamics in the formation of membrane protrusions. Trends Cell Biol..

[31] Sebé-Pedrós A., Burkhardt P., Sánchez-Pons N., Fairclough S.R., Lang B.F., King N., Ruiz-Trillo I. (2013). Insights into the origin of metazoan filopodia and microvilli. Mol. Biol. Evol..

[32] Rodríguez-García R., Volkov V.A., Chen C.-Y., Katrukha E.A., Olieric N., Aher A., Grigoriev I., López M.P., Steinmetz M.O., Kapitein L.C. (2020). Mechanisms of motor-independent membrane remodeling driven by dynamic microtubules. Curr. Biol..

[33] Kučera O., Janda D., Siahaan V., Dijkstra S.H., Pilátová E., Zatecka E., Diez S., Braun M., Lansky Z. (2020). Anillin propels myosin-independent constriction of actin rings. bioRxiv.

[34] Kreimer G. (2008). The green algal eyespot apparatus: a primordial visual system and more?. Curr. Genet..

[35] Misumi O., Matsuzaki M., Nozaki H., Miyagishima S.-Y., Mori T., Nishida K., Yagisawa F., Yoshida Y., Kuroiwa H., Kuroiwa T. (2005). *Cyanidioschyzon Merolae* Genome. A tool for facilitating comparable studies on organelle biogenesis in photosynthetic eukaryotes. Plant Physiol..

[36] Spurr A.R. (1969). A low-viscosity epoxy resin embedding medium for electron microscopy. J. Ultrastruct. Res..

[37] Dyballa N., Metzger S. (2009). fast and sensitive colloidal coomassie g-250 staining for proteins in polyacrylamide gels. J. Vis. Exp..

[38] Altschul S.F., Gish W., Miller W., Myers E.W., Lipman D.J. (1990). Basic local alignment search tool. J. Mol. Biol..

